# Development and verification of prediction models for preventing cardiovascular diseases

**DOI:** 10.1371/journal.pone.0222809

**Published:** 2019-09-19

**Authors:** Ji Min Sung, In-Jeong Cho, David Sung, Sunhee Kim, Hyeon Chang Kim, Myeong-Hun Chae, Maryam Kavousi, Oscar L. Rueda-Ochoa, M. Arfan Ikram, Oscar H. Franco, Hyuk-Jae Chang

**Affiliations:** 1 Integrative Research Center for Cerebrovascular and Cardiovascular diseases, Yonsei University College of Medicine, Yonsei University Health System, Seoul, Korea; 2 Division of Cardiology, Ewha University College of Medicine, Seoul, Korea; 3 Data Science Team of KT NexR, Seoul, Korea; 4 Yonsei University College of Medicine, Yonsei University Health System, Seoul, Korea; 5 Division of Cardiology, Severance Cardiovascular Hospital, Yonsei University College of Medicine, Seoul, Korea; 6 Department of Preventive Medicine, Yonsei University College of Medicine, Seoul, Korea; 7 AI R&D Lab. of Selvas AI Inc., Seoul, Korea; 8 Department of Epidemiology, Erasmus MC, Rotterdam, the Netherlands; 9 School of Medicine, Faculty of Health, Universidad Industrial de Santander UIS, Bucaramanga, Colombia; 10 Department of Radiology, Erasmus MC, Rotterdam, the Netherlands; 11 Severance Biomedical Science Institute, Yonsei University College of Medicine, Seoul, Korea; University of Bologna, ITALY

## Abstract

**Objectives:**

Cardiovascular disease (CVD) is one of the major causes of death worldwide. For improved accuracy of CVD prediction, risk classification was performed using national time-series health examination data. The data offers an opportunity to access deep learning (RNN-LSTM), which is widely known as an outstanding algorithm for analyzing time-series datasets. The objective of this study was to show the improved accuracy of deep learning by comparing the performance of a Cox hazard regression and RNN-LSTM based on survival analysis.

**Methods and findings:**

We selected 361,239 subjects (age 40 to 79 years) with more than two health examination records from 2002–2006 using the National Health Insurance System-National Health Screening Cohort (NHIS-HEALS). The average number of health screenings (from 2002–2013) used in the analysis was 2.9 ± 1.0. Two CVD prediction models were developed from the NHIS-HEALS data: a Cox hazard regression model and a deep learning model. In an internal validation of the NHIS-HEALS dataset, the Cox regression model showed a highest time-dependent area under the curve (AUC) of 0.79 (95% CI 0.70 to 0.87) for in females and 0.75 (95% CI 0.70 to 0.80) in males at 2 years. The deep learning model showed a highest time-dependent AUC of 0.94 (95% CI 0.91 to 0.97) for in females and 0.96 (95% CI 0.95 to 0.97) in males at 2 years. Layer-wise Relevance Propagation (LRP) revealed that age was the variable that had the greatest effect on CVD, followed by systolic blood pressure (SBP) and diastolic blood pressure (DBP), in that order.

**Conclusion:**

The performance of the deep learning model for predicting CVD occurrences was better than that of the Cox regression model. In addition, it was confirmed that the known risk factors shown to be important by previous clinical studies were extracted from the study results using LRP.

## Introduction

Cardiovascular disease (CVD) is one of the leading causes of mortality worldwide [1]. Because multiple risk factors are associated with CVD, managing these risk factors is difficult but could prevent numerous deaths. In previous studies, various prediction models were developed to identify individuals that have a high risk of developing CVD, and Cox hazard regression analysis has been the traditional approach [2–7]. Cox hazard regression models have been used to identify risk factors in phases of risk ratios and provide a probability that an individual will develop CVD, enabling personalized treatment for high-risk individuals [8].

Cox hazard regression models assume the independence of predictors using pre-specified risk factors [8]. In a prospective cohort, the selected risk factors are measured at pre-planned times, so information on the collected risk factors can be fully used by statistical methods. However, due to the variety of types and cycles of risk factor measurements in clinical studies, existing statistical models do not have all the information on CVD risk, and only parts of those databases are available. The modern hospital information system (HIS) has created complex, digitalized, time-series health dataset. However, appropriate analysis methods for maximizing the predictive performance using these multi-measurement datasets have not been clearly defined.

Deep learning is a type of machine learning algorithm [9,10] and has been demonstrated to have outstanding performance capabilities for classification of data [11,12]. The overall transformations involve multiple layers in deep learning [8], which can improve a predictive model’s performance in analyzing datasets composed of complex time-varying data. To date, several small studies have explored the potential of deep learning for disease–risk prediction using data from specific time points [13–15]. Accordingly, this study attempts to evaluate the discriminative accuracy of a deep learning algorithm model, based on survival analysis with repeated health data for CVD prediction, by comparing the results with a conventional Cox hazard regression analysis. The forecasts for the two models were calculated for a specific time point through classification. We also verified the models.

## Methods

### Data source

This study used the National Health Insurance System-National Health Screening Cohort (NHIS-HEALS) [16] data derived from a national health screening program and the national health insurance claim database in the National Health Insurance System (NHIS) of South Korea and prospective cohort data from the Rotterdam Study [17]. Data from the NHIS-HEALS was fully anonymized for all analyses and informed consent was not specifically obtained from each participant. In the Rotterdam Study, all data were collected in a standardized manner according to a pre-determined study protocol and informed consent was obtained from all participants. This study was approved and exempt from informed consent by the Institutional Review Board of Yonsei University, Severance Hospital in Seoul, South Korea (IRB no.4-2016-0383).

### Study population

The NHIS constructed the NHIS-HEALS cohort, which consists of data from 514,866 people (age 40 to 79 years), randomly sampled from 10% of the source population, who had undergone the NHIS health examination in 2002–2003 as the baseline. This cohort data represents the Korean adult population, as every Korean over 40 years of age is required to join the NHIS and is recommended to have regular biennial checkups. Due to this recommendation, the baseline for this study can be defined as the year 2002–2003. The data includes information from 2002 to 2013, and repeated data measurements were selected for research purposes as repeated data measurements are useful for identifying discriminative accuracy.

The following steps were implemented for the data manipulation: (a) out of 514,866 individuals, except those with pre-existing histories of CVD; (b) those who had treatment records of CVD or death, or a history of stroke or heart disease at the baseline were removed; (c) only those with more than two screenings from 2002–2006 were included; and (d) the remaining group, 361,239 subjects, who did not have CVD at the baseline were divided into two subgroups; a training set (80%, 288,992 subjects) and a test set (20%, 72,247subjects).

**Fig 1:**
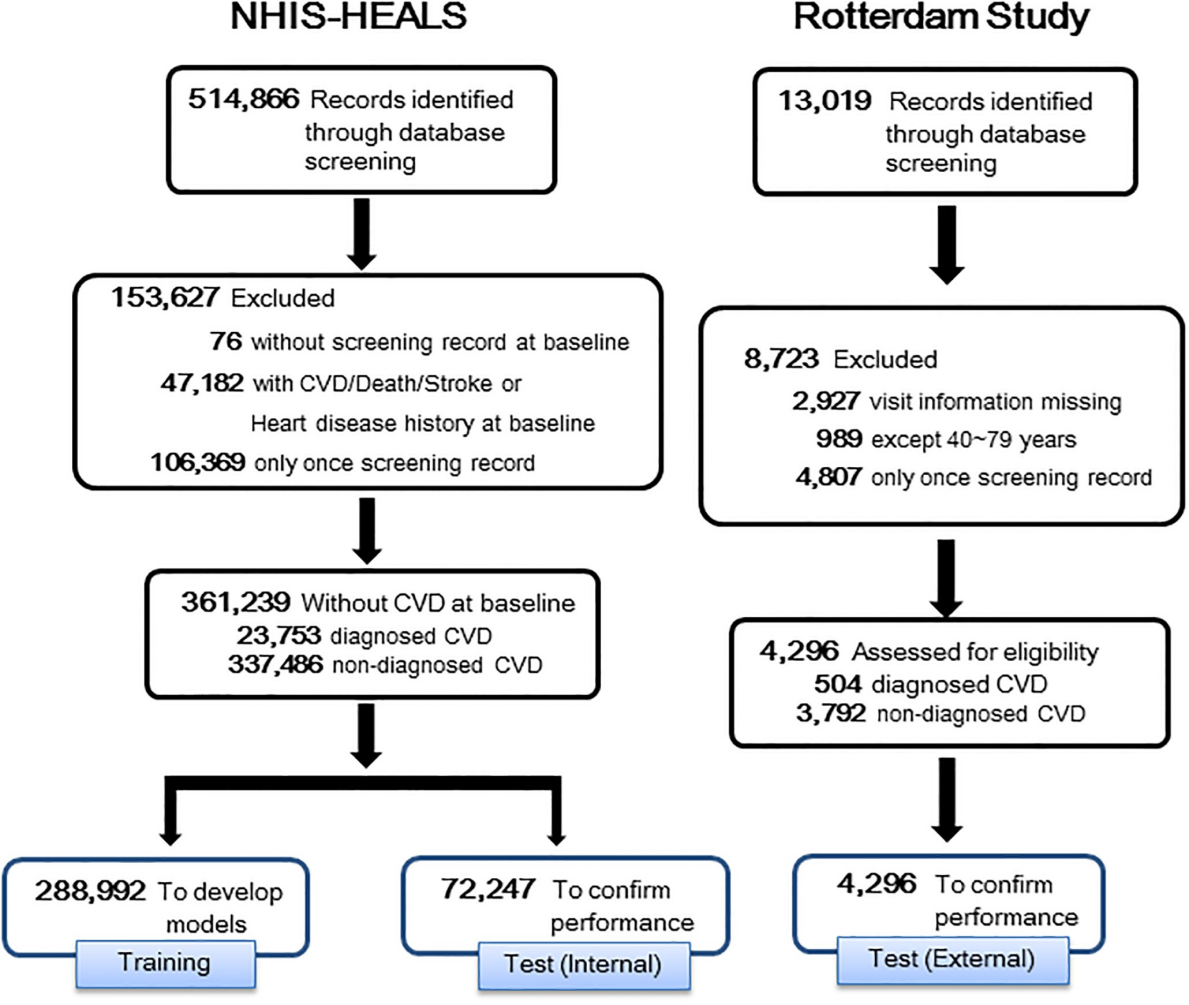
The process for selecting study subjects.

Consequently, a total of 288,992 subjects were allocated to the training set (18,904 with CVD vs. 277,088 without CVD) and were utilized for building a separate model for gender. Also, we constructed a specific dataset for the external verification of the Rotterdam Study, to verify the performance of the model that was built by NHIS-HEALS (See S1 Appendix for the details of the Rotterdam Study). For the external verification, the Rotterdam Study has been constructed based on the same criteria as the training set utilizing the NHIS-HEALS cohort data. Fig 1 presents the flow and detailed processes of all data handling.

### Outcomes

The primary outcome was defined as the occurrence of one of the following events during the follow-up period after the baseline health examination: (1) death from CVD (International Classification of Diseases 10th edition [ICD-10] codes), (2) hospitalization due to myocardial infarction, coronary arterial intervention or bypass surgery or (3) hospitalization due to stroke.

### Converting the output variables for clinical studies

In the field of medical research, we need to determine how to use Recurrent Neural Network-Long Short-Term Memory (RNN-LSTM) based on survival analysis to determine whether disease occurred at a specific time point. Thus, we transformed the binary output variable into multiple time point output variable vectors for developing point-in-time analysis according to previous studies utilizing vector variables [18–21].

To find the specific points-in-time when diseases occurred, we analyzed each year’s case by converting the output variables. In the output layer, each node represents a time interval, from two to ten years, in 1-year intervals. The value of each node is the probability of survival for that point-in-time. The survival probability after disease initiation is 0, and the probability of disease after the disease-free survival time for censored cases is presumed by the Kaplan-Meier survival function [20]. This predicted output is the probability of survival for each time point.

Based on the predictive results of the deep learning algorithm, we compared the survival probability from the Cox regression and the probability from the deep learning model with the correct answers to confirm the AUC for each year. Thus, we demonstrated the predictive performance of our models, Cox regression and deep learning, by calculating the AUC for each year.

### Risk predictors used in model building

To develop the risk model, an *a priori* decision was made that assumed the following variables—age, body mass index (BMI), systolic blood pressure (SBP), diastolic blood pressure (DBP), total cholesterol (TC), fasting plasma glucose (FPG), current smoking and exercise—were predictor variables. Details of the variables included in Cox regression and deep learning models are described in S1 Table. Variables with missing data (less than 4%) were included in the analysis. In cases where the data was missing, multiple imputations by fully conditional specifications [22] were performed using the following MI procedure in SAS 9.4 [23].

### Prediction model of statistics and deep learning

We developed CVD prediction models by sex, as it is known that there are significant differences in the risk factors and occurrence rates of CVD between the sexes [24]. Data from the baseline health examinations and repeated measurements from the periodic follow-up examinations were used to build the prediction models. The time to event was defined as the time between the date of the first health examination and that of the first diagnosed event or the last date in the cohort in non-event subjects. Also, the data used in the analysis was the health examination data from 2002–2006. For example, if a patient with a disease in 2005 had two records of health screenings in 2002 and 2004, the analysis was performed using both health screening records. As another example, assuming that a patient diagnosed with a disease in 2009 had four health examinations every two years from 2002 to 2008, the analysis was conducted by using only health information from 2002 to 2006. This decision was made to control the disparity in the volume of information among subjects by adjusting the amount of time from which screening records were used.

First of all, the Cox model using longitudinal data and its improved accuracy over single-measure methods have been described previously in order to compare it with deep learning using longitudinal data [25]. In this study, for the Cox regression model, we used the mean, minimum and maximum values and standard deviations (SDs) as continuous variables and the mean and SDs as categorical variables calculated from the periodic health screening data. The details of the measurement of risk factors in the Cox modeling are described in S2 Appendix.

For the deep learning algorithm model based on survival analysis, an RNN-LSTM [26] network was used. The deep learning algorithm was constructed using the same variables used in the Cox regression model with longitudinal data. Our proposed LSTM model was designed with the following structure. For the optimization of the algorithm, RMSProp [27] was used to update the parameters through back-propagation. Hyper-parameters at a learning rate of 0.01 were configured, with a dropout probability of 50%, and a mini-batch of 64. The correct answer was one-hot encoded to be used for cross-entropy in a loss function. The number of classes was 2. The details of the deep learning and model building process are demonstrated in S3 Appendix. Then, the calculated performance metrics were evaluated with C-statistics or AUC [28]. Research has demonstrated that C-statistics is analogous to AUC [29].

### Evaluation of prediction performance

The prediction performances of each prediction model were evaluated using NHIS-HEALS data and external test data, Rotterdam Study. Model discrimination was quantified by calculating the C-statistics for the survival model. All statistical analyses were conducted with SAS (version 9.4, SAS Inc., Cary, NC, USA) and the R Statistical Package (www.R-project.org). The statistical significance criterion was set at 2-sided p < 0.05.

### The solution to the problem of understanding classification decisions

In order to overcome the problem which was the inability to explain the reason for classification, we confirmed the influence of the input variables using a Layer-wise Relevance Propagation (LRP) [30], one of many explainable artificial intelligence (XAI) techniques used in artificial neural networks [31, 32].

The order of each variable is the mean of the LRP output values for each input sample, which are sorted in descending order. The number of feature variables is n, the number of input samples is m, and the output value of the prediction model is o = {o_1_ … o_m_}, thus, the ranking of feature variables is expressed as follows.

$$rank(o)\;=\;desc\_sort\;(\frac{1}{m}\;\sum_{i\;=\;0}^{n}\sum_{j\;=\;0}^{m}{|lrp}_{i}(o_{j})|)$$

Through this technique, we present the effect of the feature variables used to build the model.

## Results

Table 1 presents the characteristics of the training cohort at baseline. The mean age was 51.2 ± 8.9 years, and a total of 164,024 male subjects (56.76%) were included in the cohort. The average number of health screenings used in the analysis was 3.1 ± 1.1 for male subjects, and 2.6 ± 0.9 for female subjects.

**Table 1:** Baseline characteristics of the training set.

Variable	Training set	
	Male (n = 164,024)	Female (n = 124,968)
Age, years	51.2 ± 8.9	52.8 ± 9.2
Current smoking, n (%)	90,677 (55.28)	3,582 (2.87)
Exercise, n (%)	82,851 (50.51)	40,903 (32.73)
Alcohol intake, n (%)	107,962 (65.82)	21,741 (17.40)
Body mass index, kg/m^2^	24.0 ± 2.8	23.9 ± 3.0
Systolic blood pressure, mmHg	128.2 ± 17.1	124.2 ± 18.3
Diastolic blood pressure, mmHg	81.2 ± 11.3	77.3 ± 11.6
Fasting plasma glucose, mg/dL	99.1 ± 34.4	94.8 ± 31.0
Total cholesterol, mg/dL	199.0 ± 37.8	201.7 ± 39.1
Hemoglobin, g/dL	14.8 ± 1.1	12.9 ± 1.2
Aspartate transaminase, U/L	29.0 ± 19.5	24.1 ± 14.1
Alanine transaminase, U/L	30.1 ± 23.2	21.1 ± 17.1
Gamma-glutamyl transpeptidase, U/L	50.1 ± 63.2	20.9 ± 22.2
Urine protein, n (%)	2,781 (1.70)	2,192 (1.75)
History, n (%)		
Diabetes mellitus	5,989 (3.65)	4,008 (3.21)
Hypertension	8,919 (5.44)	9,715 (7.77)
Etc (include cancer)	16,841 (10.27)	12,262 (9.81)
Family history, n (%)		
Stroke	9,565 (5.83)	6,262 (5.01)
Diabetes mellitus	10,185 (6.21)	7.985 (6.39)
Heart disease	4,614 (2.81)	3,367 (2.69)
Hypertension	12,345 (7.53)	10,925 (8.74)
Etc (include cancer)	23,328 (14.22)	18,966 (15.18)
Number of periodic health examinations	3.1 ± 1.1	2.6 ± 0.9

In the internal validation using the NHIS-HEALS cohort data, the Cox regression model showed the highest time-dependent AUC was 0.79 (95% CI 0.70 to 0.87) at 2 years in female subjects. The time-dependent AUC from 3 to 7 years was around 0.7. The deep learning model showed the highest time-dependent AUC was 0.96 (95% CI 0.95 to 0.97) at 2 years in male subjects. The time-dependent AUC from 3 to 5 years was around 0.8. The remaining results are presented in S2 Table. In the external validation using data from the Rotterdam Study, the Cox regression model showed the highest time-dependent AUC was 0.73 (95% CI 0.69 to 0.76) at 8 years in female subjects. The time-dependent AUC of 3 to 10 years was around 0.7. The deep learning model showed the highest time-dependent AUC was 0.90 (95% CI 0.85 to 0.95) at 2 years in female subjects. The time-dependent AUC from 3 to 8 years was around 0.85. The remaining results are presented in S3 Table.

Furthermore, the results of the LRP demonstrated that the known risk factors identified in previous studies do affect CVD and provided numerical impact for each risk factor used in the deep learning modeling. The deep learning model showed that age was the variable that had the greatest effect on CVD occurrence. Moreover, SBP, DBP, sex and FPG were ranked at the upper. The details are described in Table 2.

**Table 2:** Rank of risk factors in deep learning model.

Feature name	Sum of ranks	Feature name	Mean of values
Age	233,322	Age	0.405
Systolic blood pressure	359,881	Systolic blood pressure	0.262
Sex	390,006	Diastolic blood pressure	0.153
Diastolic blood pressure	548,049	Sex	0.116
Fasting plasma glucose	584,936	Fasting plasma glucose	0.111
Gamma-glutamyl transpeptidase	664,941	Current smoking	0.111
Aspartate transaminase	668,470	Exercise	0.105
Hemoglobin	683,408	Aspartate transaminase	0.074
Total cholesterol	757,839	Gamma-glutamyl transpeptidase	0.066
Exercise	776,784	Hemoglobin	0.061
Alcohol intake	814,943	Alcohol intake	0.052
Body mass index	837,221	Total cholesterol	0.045
Urine protein	867,499	Body mass index	0.032
Alanine transaminase	973,370	Urine protein	0.028
Family history of etc (include cancer)	1,150,578	History of Hypertension	0.026
Family history of Stroke	1,187,298	Family history of etc (include cancer)	0.025
Family history of Diabetes mellitus	1,299,493	History of etc (include cancer)	0.022
Family history of Heart disease	1,392,376	Alanine transaminase	0.015
Family history of Hypertension	1,412,000	History of Diabetes mellitus	0.012
Current smoking	1,486,150	Family history of Hypertension	0.004
History of Hypertension	1,546,467	Family history of Diabetes mellitus	0.002
History of Diabetes mellitus	1,567,078	Family history of Stroke	0.001
History of etc (include cancer)	1,585,386	Family history of Heart disease	0.000

Sum of ranks indicate ranking each sample by absolute value of LRP, then ascending order by summing the ranks by variables in all samples. Mean of values indicate calculate the mean for the absolute value of LRP by variable in all samples and sort in descending order.

## Discussion

The principal findings of this study were as follows: (1) deep learning algorithms have significantly improved predictive power for CVD compared to Cox regression analysis. However, while the deep learning algorithm maintained high predictive power within 5 years, after that it decreased sharply. (2) The results of the verification using the Rotterdam Study confirmed that the predictive power of the deep learning algorithm compared to the Cox regression analysis was improved. This is the first large-scale and systematic assessment of a deep learning approach for predicting the occurrence of CVD at a particular point in time, suggesting that it can be generalized without racial influence. (3) The effects of the various risk factors were identified through the LRP. The LRP might be useful for identifying the impact of risk factors that the deep learning approach cannot identify.

Since the electronic health records (EHR) were introduced decades ago, huge amounts of medical data have accumulated. The nationwide repeated health screening systems in Korea cannot be applied to all medical systems, but as HIS has developed into a medical platform, the accumulation of large-scale datasets in the medical field is accelerating. The deep learning model can be a useful tool for the prediction of risk in the EHR era by providing discrimination and calibration using repeatedly measured data.

Disease prediction studies using deep learning, a subfield of machine learning, have already been studied previously [33–34] and have been shown to have high value in the classification of problems [11–12, 35–36]. Deep learning differs from statistics by Cox regression analysis. The Cox regression model assumes an independence between predefined variables and does not reflect changes in those variables over time, but the advantage of deep learning is that it can use variables that are constantly changing. As a result of this research, these advantages were identified by improving the accuracy of CVD predictions, but after five years, the performance of this model was similar to that of the Cox model. The Rotterdam Study maintains a high level of deep learning performance (an AUC of about 0.8) over a longer period of time than the Cox model. This seems to be due to an increase in CVD incidence rates over time. The reason is that the annual incidence rate of CVD in the internal data increased by about 0.5%, but in the Rotterdam Study it increased by about 1.5% and the increase rate decreases markedly from 9 year. When the rate of increase of CVD occurrence is significantly reduced, the predictive power of the deep learning model was reduced. Therefore, while deep learning is appropriate for identifying risk factors that predict the occurrence of disease within 5 years using constantly changing data after 5 years predictions require scrutiny. One of the major disadvantages of the deep learning model is that it can’t provide specific recommendations for controlling risk factors because the risk factors that affect the event occurrence are unknown. To overcome these shortcomings, we used LRP to assess the risk factors individually. The results of the LRP show that the risk factors considered to be important in previous clinical studies were similar to those shown to be important by the deep learning model: Age, gender, SBP, TC, smoking, exercise, etc [37–39].

However, this study has several limitations. First, because only the information obtained from the screening data is available, it is not possible to reflect changes in the level of risk due to unpredictable drugs or non-pharmacological treatments based on physician or patient behavior during follow-up. In addition, the risk of CVD may change due to changes in the risk factors and the interaction between risk factors, but the research on this is still lacking. Second, although we ranked the risk factors separately using LRP, the model does not know the size of the effect of the risk factors, such as the hazard ratio, due to the nature of the hidden layer of the neural network models. Therefore, further studies are needed to overcome this, as it is not yet ready for clinical use. Third, unlike the NHIS-HEALS, in the Rotterdam Study, there were limitations to the comparison of variables to the performance in the internal validation because the variables were only: age, sex, BLDS, BMI, SBP, DBP, exercise and smocking.

## Conclusions

Deep learning models have greater predictive power for CVD occurrence than the Cox regression model within five years. In addition, it was confirmed that the risk factors shown to be important in previous clinical studies were also extracted from the results of this study using LRP.

## Supporting information

S1 Appendix The Rotterdam Study design. (PDF)

S2 Appendix Methods for risk factor measurement. (PDF)

S3 Appendix Model building and training in the recurrent neural network. (PDF)

S1 Table Variables used in each prediction model. (PDF)

S2 Table Predictive performance by year and sex for the Cox regression model and deep learning model in the internal validation set. (PDF)

S3 Table Predictive performance by year and sex for the Cox regression model and deep learning model in the external validation set. (PDF)

S4 Table C-index by year and sex for Cox regression model. (PDF)

S1 Fig Calibration. The left-hand figures represent 5-years and 10-years for the Cox regression model. The right-hand figures represent 5-years and 10-years for the DL model.
(PDF)

S2 Fig AUC by year and sex for the Cox regression model and deep learning model. (PDF)

## References

[1] Ezzati M, Vander Hoorn S, Lawes CM, Leach R, James WP, Lopez AD, Rodgers A, Murray CJ. Rethinking the "diseases of affluence" paradigm: global patterns of nutritional risks in relation to economic development. PLoS Med. 2005 5;2(5):e133 10.1371/journal.pmed.0020133 15916467 PMC1088287

[2] Conroy RM, Pyorala K, Fitzgerald AP, Sans S, Menotti A, De Backer G, De Bacquer D, Ducimetiere P, Jousilahti P, Keil U, Njolstad I, Oganov RG, Thomsen T, Tunstall-Pedoe H, Tverdal A, Wedel H, Whincup P, Wilhelmsen L, Graham IM. Estimation of ten-year risk of fatal cardiovascular disease in Europe: the SCORE project. Eur Heart J. 2003;24:987–1003. 10.1016/s0195-668x(03)00114-3 12788299

[3] Hippisley-Cox J, Coupland C, Vinogradova Y, Robson J, May M, Brindle P. Derivation and validation of QRISK, a new cardiovascular disease risk score for the United Kingdom: prospective open cohort study. Bmj. 2007;335:136 10.1136/bmj.39261.471806.55 17615182 PMC1925200

[4] D’Agostino RB Sr., Grundy S, Sullivan LM, Wilson P. Validation of the Framingham coronary heart disease prediction scores: results of a multiple ethnic groups investigation. Jama. 2001;286:180–187. 10.1001/jama.286.2.180 11448281

[5] Lloyd-Jones DM, Leip EP, Larson MG, D’Agostino RB, Beiser A, Wilson PW, Wolf PA, Levy D. Prediction of lifetime risk for cardiovascular disease by risk factor burden at 50 years of age. Circulation. 2006;113:791–798. 10.1161/CIRCULATIONAHA.105.548206 16461820

[6] Pencina MJ, D’Agostino RB Sr., Larson MG, Massaro JM, Vasan RS. Predicting the 30-year risk of cardiovascular disease: the framingham heart study. Circulation. 2009;119:3078–3084. 10.1161/CIRCULATIONAHA.108.816694 19506114 PMC2748236

[7] Wilson PW, D’Agostino RB, Levy D, Belanger AM, Silbershatz H, Kannel WB. Prediction of coronary heart disease using risk factor categories. Circulation. 1998;97:1837–1847. 10.1161/01.cir.97.18.1837 9603539

[8] Goldstein BA, Navar AM, Carter RE. Moving beyond regression techniques in cardiovascular risk prediction: applying machine learning to address analytic challenges. Eur Heart J. 2016;19. 10.1093/eurheartj/ehw302 PMC5837244 27436868

[9] Waljee AK, Higgins PD. Machine learning in medicine: a primer for physicians. Am J Gastroenterol. 2010;105:1224–1226. 10.1038/ajg.2010.173 20523307

[10] Deo RC. Machine Learning in Medicine. Circulation. 2015;132:1920–1930. 10.1161/CIRCULATIONAHA.115.001593 26572668 PMC5831252

[11] Dean J, Corrado G, Monga R, Chen K, Devin M, Mao M, Senior A, Tucker P, Yang K, Le QV. Large scale distributed deep networks. In. Advances in neural information processing systems 2012:1223–1231.

[12] Hinton G, Deng L, Yu D, Dahl GE, Mohamed A-r, Jaitly N, Senior A, Vanhoucke V, Nguyen, Sainath TN. Deep neural networks for acoustic modeling in speech recognition: The shared views of four research groups. IEEE Signal Processing Magazine. 2012;29:82–97.

[13] Narain R, Saxena S, Goyal AK. Cardiovascular risk prediction: a comparative study of Framingham and quantum neural network based approach. Patient Prefer Adherence. 2016;10:1259–1270. 10.2147/PPA.S108203 27486312 PMC4958363

[14] Khatibi V, Montazer GA. A fuzzy-evidential hybrid inference engine for coronary heart disease risk assessment. Expert Systems with Applications. 2010;37:8536–8542.

[15] Kukar M, Kononenko I, Grošelj C, Kralj K, Fettich J. Analysing and improving the diagnosis of ischaemic heart disease with machine learning. Artificial intelligence in medicine. 1999;16:25–50. 10225345 10.1016/s0933-3657(98)00063-3

[16] Seong SC, Kim YY, Park SK, et al Cohort profile: the National Health Insurance Service-National Health Screening Cohort (NHIS-HEALS) in Korea. BMJ Open 2017;7:e016640 10.1136/bmjopen-2017-016640 28947447 PMC5623538

[17] Hofman A, Brusselle GG, Darwish Murad S, et al The Rotterdam Study: 2016 objectives and design update. Eur J Epidemiol 2015;30:661–708. 10.1007/s10654-015-0082-x 26386597 PMC4579264

[18] Street, W. N. (1998, July). A Neural Network Model for Prognostic Prediction. In ICML (pp. 540–546).

[19] Baesens B., Van Gestel T., Stepanova M., Van den Poel D., & Vanthienen J. (2005). Neural network survival analysis for personal loan data. Journal of the Operational Research Society, 56(9), 1089–1098.,

[20] Chi, C. L., Street, W. N., & Wolberg, W. H. (2007). Application of artificial neural network-based survival analysis on two breast cancer datasets. In AMIA Annual Symposium Proceedings (Vol. 2007, p. 130). American Medical Informatics Association. PMC2813661 18693812

[21] Dezfouli, H. N., & Bakar, M. R. A. (2012, September). Feed forward neural networks models for survival analysis. In Statistics in Science, Business, and Engineering (ICSSBE), 2012 International Conference on (pp. 1–5). IEEE).

[22] Van Buuren S. Multiple imputation of discrete and continuous data by fully conditional specification. Statistical methods in medical research 2007;16:219–42. 10.1177/0962280206074463 17621469

[23] SAS INSTITUTE INC. SAS/STAT® 14.1 User’s Guide. The MI Procedure. 2015.

[24] Mosca L, Barrett-Connor E, Wenger NK. Sex/gender differences in cardiovascular disease prevention: what a difference a decade makes. Circulation. 2011;124:2145–2154. 10.1161/CIRCULATIONAHA.110.968792 . 22064958 PMC3362050

[25] Cho IJ, Sung JM, Chang HJ, et al Incremental Value of Repeated Risk Factor Measurements for Cardiovascular Disease Prediction in Middle-Aged Korean Adults: Results From the NHIS-HEALS (National Health Insurance System-National Health Screening Cohort). Circ Cardiovasc Qual Outcomes 2017;10:004197. 10.1161/CIRCOUTCOMES.117.004197 29150537

[26] Hochreiter S, Schmidhuber J. Long short-term memory. Neural computation 1997;9:1735–80. 9377276 10.1162/neco.1997.9.8.1735

[27] Tieleman T, Hinton G. Lecture 6.5-rmsprop: Divide the gradient by a running average of its recent magnitude. COURSERA: Neural networks for machine learning. 2012;4.

[28] Harrell FE Jr, Califf RM, Pryor DB, Lee KL, Rosati RA. Evaluating the yield of medical tests. JAMA.1982;247(18):2543–2546. 7069920

[29] Hanley JA, McNeil BJ. The meaning and use of the area under a receiver operating characteristic (ROC) curve. Radiology. 1982;143(1):29–36. 10.1148/radiology.143.1.7063747 7063747

[30] Bach Sebastian, et al "On pixel-wise explanations for non-linear classifier decisions by layer-wise relevance propagation." PloS one 10 7 (2015): e0130140 10.1371/journal.pone.0130140 26161953 PMC4498753

[31] Ras Gabriëlle, van Gerven Marcel, and Haselager Pim. "Explanation methods in deep learning: Users, values, concerns and challenges" Explainable and Interpretable Models in Computer Vision and Machine Learning. Springer, Cham, 2018 19–36.

[32] Arras Leila, et al "Explaining Recurrent Neural Network Predictions in Sentiment Analysis." EMNLP 2017 (2017): 159.

[33] Jarrett D, Yoon J, van der Schaar M. Dynamic Prediction in Clinical Survival Analysis using Temporal Convolutional Networks. IEEE J Biomed Health Inform. 2019. 10.1109/JBHI.2019.2929264 31331898

[34] Wang T, Qiu RG, Yu M. Predictive Modeling of the Progression of Alzheimer’s Disease with Recurrent Neural Networks. Sci Rep. 2018; 8: 9161 10.1038/s41598-018-27337-w 29907747 PMC6003986

[35] LeCun Y, Bengio Y, Hinton G. Deep learning. Nature 2015 5 28;521(7553):436–444. 10.1038/nature14539 26017442

[36] Min S, Lee B, Yoon S. Deep learning in bioinformatics. Brief Bioinform 2017 9 1;18(5):851–869. 10.1093/bib/bbw068 27473064

[37] Ruwanpathirana T, Owen A, Reid CM. Review on Cardiovascular Risk Prediction. Cardiovasc Ther. 2015 4; 33(2):62–70. 10.1111/1755-5922.12110 25758853

[38] Vikulova DN, Grubisic M, et al Premature Atherosclerotic Cardiovascular Disease: Trends in Incidence, Risk Factors, and Sex-Related Differences, 2000 to 2016. J Am Heart Assoc. 2019 7 16; 8(14):e012178 10.1161/JAHA.119.012178 31280642 PMC6662126

[39] Ambale-Venkatesh B, Yang X, et al Cardiovascular Event Prediction by Machine Learning The Multi-Ethnic Study of Atherosclerosis. Circ Res. 2017 10 13;121(9):1092–1101. 10.1161/CIRCRESAHA.117.311312 28794054 PMC5640485

