# Supplementary material for: Development and verification of prediction models for preventing cardiovascular diseases
Source: PLoS One. 2019 Sep 19;14(9):e0222809. doi: 10.1371/journal.pone.0222809 (PMC6752799; doi:10.1371/journal.pone.0222809)
Supplement: S1 Appendix — (PDF) [file pone.0222809.s001.pdf]

The Rotterdam Study (RS) is a prospective cohort study ongoing since 1990 in the city of Rotterdam in the Netherlands.[1] The cohort has been extended twice (in 2000 and in 2006). By the end of 2008, the RS comprised 14,926 individuals aged 45 years or over. The baseline examination of the current study used data from the third visit of the original cohort (RS-I-3) and the first visits of the two extended cohorts (RS-II-1, RS-III-1). The participants have been re-visited at the research center every 3-5 years. Blood pressure was measured at the right brachial artery with a random-zero sphygmomanometer with the participant in sitting position, and the mean of 2 consecutive measurements was used. Serum glucose and serum total cholesterol levels were measured with standard laboratory techniques. Information on study outcomes was gathered from general practitioners and from letters and discharge reports from medical specialists. Events were adjudicated by study physicians as described previously.[2,3] For this project, incident coronary heart disease (CHD) was defined as the occurrence of a non-fatal myocardial infarction or coronary revascularization.[2] Strokes were diagnosed when a patient had typical neurological symptoms and a computed tomography or magnetic resonance imaging, made within 4 weeks after the occurrence of stroke, confirmed the diagnosis.[3] For this study, cardiovascular mortality was based on ICD-10 codes I-00 to I-99.

Reference

1. Hofman A, Brusselle GG, Darwish Murad S, et al. The Rotterdam Study: 2016 objectives and design update. Eur J Epidemiol. 2015;30(8):661-708.

2. Leening MJ, Kavousi M, Heeringa J, et al. Methods of data collection and definitions of cardiac outcomes in the Rotterdam Study. European journal of epidemiology. 2012;27(3):173-185.

3. Wieberdink RG, Ikram MA, Hofman A, Koudstaal PJ, Breteler MM. Trends in stroke incidence rates and stroke risk factors in Rotterdam, the Netherlands from 1990 to 2008. European journal of epidemiology. 2012;27(4):287-295.
