# Supplementary material for: Development and verification of prediction models for preventing cardiovascular diseases
Source: PLoS One. 2019 Sep 19;14(9):e0222809. doi: 10.1371/journal.pone.0222809 (PMC6752799; doi:10.1371/journal.pone.0222809)
Supplement: S2 Appendix — (PDF) [file pone.0222809.s002.pdf]

Resting systolic blood pressure (SBP) and diastolic blood pressure (DBP) were measured at each health screening. Body mass index (BMI) was calculated using height and weight. Overnight fasting blood samples were collected for measurement of fasting plasma glucose (FPG) and total cholesterol (TC), aspartate aminotransferase (AST), alanine transaminase (ALT), gamma-glutamyl transferase (γ-GTP) levels. Current smoking, alcohol intake and exercise were obtained via self-reported questionnaire. These measures were graded as “yes” or “no” for current smoking status, alcohol intake and exercise. Regular exercise more than once per week was graded as “yes” for exercise. For the categorical variables of smoking, alcohol intake and exercise, presence and absence of were coded as 1 and 0, respectively. Family history of diabetes, hypertension, heart disease, stroke, etc (include cancer) were figured through the questionnaire. The family history of diseases has been defined as any family member who has been diagnosed with diseases by a physician. In addition, medical history was acquired by survey questionnaire of the health check-ups.
