# Supplementary material for: Development and verification of prediction models for preventing cardiovascular diseases
Source: PLoS One. 2019 Sep 19;14(9):e0222809. doi: 10.1371/journal.pone.0222809 (PMC6752799; doi:10.1371/journal.pone.0222809)
Supplement: S3 Appendix — (PDF) [file pone.0222809.s003.pdf]

RNN-LSTM was developed to solve long-range dependency and vanishing gradient problems seen in RNN, which degraded performance as event length increased. Our proposed LSTM model is designed with the following structure. Our proposed LSTM model is designed with the following structure. For the optimization of the algorithm, RMSProp[1] was used to update parameters through back-propagation. The learning sample set $\mathbb{S}$ is consisted of ordered pairs $(\mathbf{x}, \mathbf{y})$ with inputs and correct answers. Input x is an element of input set $\mathbb{X}$, whereas correct answer $\mathbf{y}$ is an element of correct answers set $\mathbb{Y}$. Here, $\mathbf{x}$ is consisted of serial data in the form of an individual's information. The T in input $\mathbf{x} = (\mathbf{x}_1, \dots, \mathbf{x}_\mathrm{t}, \dots, \mathbf{x}_\mathrm{T})$ represents the length of a sample (i.e. the number of events) and varies individually. A health examination record is consisted of an event; and, all events $\mathbf{x}_1, \dots, \mathbf{x}_\mathrm{T}$ for a person are in chronological order. x_t represents an event (examination record) at a specific time and is used as a vector with features. The correct answer $\mathbf{y} = (\mathrm{y}_1, \dots, \mathrm{y}_\mathrm{K})$ has a Boolean value indicating whether or not CVD occurred in the past. Hyper-parameters at a learning rate of 0.01 were configured, a dropout probability of 50%, and a mini-batch of 64. The correct answer is one-hot encoded to be used as cross entropy in a loss function. K—the number of classes—is set as 2. Assuming the output of prediction model is $\hat{\mathrm{y}} = (\hat{\mathrm{y}}_1, \dots, \hat{\mathrm{y}}_K)$, cross entropy as shown in Equation 1 is used for our loss function.

$$\mathrm{E}(y, \hat{y}) = -\sum_{i=1}^{K} [y_i \log(\hat{y}_i) + (1 - y_i)\log(1 - \hat{y}_i)] \qquad \text{(Equation 1)}$$

The output sequence for Long Short-Term Memory (LSTM) was $\mathbf{o} = (\mathbf{o}_1, \dots \mathbf{o}_\mathrm{t}, \dots, \mathbf{o}_\mathrm{T})$ where T was the event length being the same as the event length of x. The prediction result of an input sample provided the probability of CVD in the near future taken the occurrence of past event into account.

Only the last output $\mathbf{o}_\mathrm{T}$ among $\mathbf{o}_1, \dots \mathbf{o}_\mathrm{t}, \dots, \mathbf{o}_\mathrm{T}$ was used and reflected to an output z as shown in Equation 2. Here, $\mathrm{W} \in \mathbb{R}^{K \times H}$ was the parameter to be optimized and H was the number of hidden nodes in the last hidden layer. To calculate the probability of $\hat{y}_i$ from z, the softmax function was used as shown in Equation 3.

$$\mathrm{z} = \mathrm{W}\mathbf{o}_\mathrm{T} \qquad \text{(Equation 2)}$$

$$\hat{y}_\mathrm{i} = \frac{e^{z_i}}{\sum_{j=1}^{K} e^{z_j}} \quad \text{for } i = 1, \dots, K \qquad \text{(Equation 3)}$$

LSTM had a memory cell with input, forget and output gates. Each LSTM unit uses the equations in Equation 4 which are commonly used in LSTM.

$$i_t = \sigma(W_{xi}x_t + W_{hi}h_{t-1} + W_{ci} \circ c_{t-1} + b_i)$$

$$f_t = \sigma\left(W_{xf}x_t + W_{hf}h_{t-1} + W_{cf} \circ c_{t-1} + b_f\right)$$

$$c_t = f_t \circ c_{t-1} + i_t \circ \tanh(W_{xc}x_t + W_{hc}h_{t-1} + b_c \quad)$$

$$o_t = \sigma(W_{xo}x_t + W_{ho}h_{t-1} + W_{co} \circ c_t + b_o)$$

$$h_t = o_t \circ \tanh(c_t) \qquad \text{(Equation 4)}$$

σ was the logistic sigmoid function and i, f, o and c were respectively the *input gate*, *forget gate*, *output gate*, and *cell*. The subscripts in weight matrix above have an obvious meaning. For example, W_{hi} is the hidden-input gate matrix while W_{xo} being the input-output gate matrix. The bs are bias terms which are added for i, f, o and c equations. Let N be the number of LSTM blocks and M the number of inputs, $W_i, W_f, W_c, W_o \in \mathbb{R}^{N\times M}$.

Reference

1. Tieleman T, Hinton G. Lecture 6.5-rmsprop: Divide the gradient by a running average of its recent magnitude. COURSERA: Neural networks for machine learning. 2012;4.
