# Supplementary material for: Development and verification of prediction models for preventing cardiovascular diseases
Source: PLoS One. 2019 Sep 19;14(9):e0222809. doi: 10.1371/journal.pone.0222809 (PMC6752799; doi:10.1371/journal.pone.0222809)
Supplement: S1 Table — (PDF) [file pone.0222809.s004.pdf]

| Model | Variables |
|---|---|
| Cox regression | Age at baseline |
| | Mean, standard deviation, minimum value, and maximum value of Body mass index, Systolic blood pressure, Diastolic blood pressure, Fasting plasma glucose and Total cholesterol, Hemoglobin, Aspartate transaminase, Alanine transaminase, Gamma-glutamyl transpeptidase |
| | Mean and standard deviation of Current smoking and Exercise, Alcohol intake, Urine protein |
| | Mean of Family history of Hypertension, Heart disease, Stroke, Diabetes mellitus, etc(include cancer) and history Hypertension, Diabetes mellitus, etc(include cancer) |
| Deep learning | Date of each health examination |
| | Age at baseline |
| | Body mass index, Systolic blood pressure, Diastolic blood pressure, Fasting plasma glucose, Total cholesterol, Hemoglobin, Aspartate transaminase, Alanine transaminase, Gamma-glutamyl transpeptidase, Current smoking, Exercise, Alcohol intake, Urine protein, Family history of Hypertension, Heart disease, Stroke, Diabetes mellitus, Etc(include cancer) and History Hypertension, Diabetes mellitus, etc(include cancer) |
