# Supplementary material for: Development and verification of prediction models for preventing cardiovascular diseases
Source: PLoS One. 2019 Sep 19;14(9):e0222809. doi: 10.1371/journal.pone.0222809 (PMC6752799; doi:10.1371/journal.pone.0222809)
Supplement: S2 Table — (PDF) [file pone.0222809.s005.pdf]

(a) Male

COX	AUC	Sensitivity	Specificity	Accuracy	PPV	NPV
2year	0.753(0.703-0.803)	71.8 (61.4, 82.3)	68.2 (67.8, 68.7)	68.2 (68.2, 68.2)	0.4 (0.3, 0.5)	99.9 (99.9, 100)
3year	0.742(0.716-0.769)	85.9 (81.9, 89.8)	49.2 (48.7, 49.6)	49.4 (49.4, 49.4)	1.2 (1.1, 1.4)	99.8 (99.7, 99.8)
4year	0.750(0.731-0.768)	77.4 (74.1, 80.0)	60.2 (59.7, 60.6)	60.4 (60.4, 60.4)	2.8 (2.6, 3.1)	99.4 (99.3, 99.5)
5year	0.760(0.745-0.774)	78.4 (75.7, 81.0)	60.7 (60.2, 61.2)	61.1 (61.1, 61.1)	4.5 (4.2, 4.8)	99.2 (99.1, 99.3)
6year	0.761(0.748-0.773)	74.4 (72.1, 76.8)	65.2 (64.7, 65.7)	65.5 (65.5, 65.5)	6.5 (6.1, 6.9)	98.7 (98.6, 98.9)
7year	0.754(0.743-0.765)	72.8 (70.6, 74.9)	65.5 (65.0, 66.0)	65.8 (65.8, 65.8)	8.2 (7.7, 8.6)	98.3 (98.1, 98.4)
8year	0.753(0.743-0.764)	72.2 (70.2, 74.1)	65.8 (65.3, 66.3)	66.1 (66.1, 66.1)	9.8 (9.4, 10.3)	97.9 (97.7, 98.0)
9year	0.753(0.743-0.763)	71.1 (69.3, 72.9)	66.5 (66.0, 67.0)	66.8 (66.8, 66.8)	11.6 (11.1, 12.2)	97.4 (97.2, 97.6)
10year	0.750(0.741-0.759)	70.3 (68.6, 72.0)	67.1 (66.6, 67.6)	67.3 (67.3, 67.3)	13.4 (12.8, 13.9)	96.9 (96.7, 97.1)
**DL**						
2year	0.96 (0.95-0.97)	97.06 (93.78, 100.34)	87.66 (87.34, 87.97)	87.68 (87.36, 88.00)	1.92 (1.55, 2.30)	99.99 (99.98, 100)
3year	0.88 (0.87-0.89)	94.68 (92.61, 96.75)	67.22 (66.76, 67.68)	67.52 (67.07, 67.97)	3.11 (2.82, 3.40)	99.91 (99.88, 99.95)
4year	0.84 (0.82-0.85)	87.80 (85.68, 89.92)	63.67 (63.20, 64.14)	64.21 (63.74, 64.67)	5.24 (4.89, 5.59)	99.56 (99.48, 99.64)
5year	0.80 (0.79-0.81)	78.70 (76.60, 80.80)	65.73 (65.26, 66.20)	66.19 (65.73, 66.65)	7.81 (7.37, 8.24)	98.82 (98.69, 98.95)
6year	0.78 (0.77-0.79)	73.58 (71.65, 75.52)	69.58 (69.12, 70.03)	69.77 (69.33, 70.22)	11.00 (10.46, 11.53)	98.10 (97.94, 98.26)
7year	0.77 (0.76-0.78)	73.38 (71.66, 75.09)	68.24 (67.77, 68.70)	68.56 (68.11, 69.01)	13.31 (12.75, 13.87)	97.47 (97.29, 97.66)
8year	0.77 (0.76-0.78)	72.31 (70.75, 73.88)	68.89 (68.42, 69.36)	69.15 (68.71, 69.60)	16.12 (15.51, 16.73)	96.78 (96.57, 96.99)
9year	0.77 (0.76-0.78)	72.49 (71.06, 73.92)	67.99 (67.51, 68.46)	68.40 (67.95, 68.85)	18.53 (17.91, 19.16)	96.09 (95.86, 96.33)
10year	0.77 (0.76-0.78)	70.55 (69.20, 71.91)	70.43 (69.97, 70.90)	70.45 (70.00, 70.89)	22.11 (21.42, 22.80)	95.26 (95.01, 95.52)

(b) Female

COX	AUC	Sensitivity	Specificity	Accuracy	PPV	NPV
2year	0.789(0.704-0.874)	67.7 (51.3, 84.2)	81.2 (80.7, 81.6)	81.2 (81.2, 81.2)	0.4 (0.2, 0.5)	100 (99.9, 100)
3year	0.784(0.750-0.818)	68.4 (61.1 75.6)	73.5 (73.0, 74.0)	73.5 (73.5, 73.5)	1.3 (1.1, 1.5)	99.8 (99.7, 99.8)
4year	0.784(0.758-0.809)	68.5 (63.3, 73.6)	73.7 (73.2, 74.2)	73.7 (73.7, 73.7)	2.6 (2.3, 2.9)	99.6 (99.5, 99.6)
5year	0.783(0.762-0.804)	63.7 (59.4, 68.0)	79.6 (79.2, 80.1)	79.4 (79.4, 79.4)	4.7 (4.2, 5.2)	99.3 (99.2, 99.4)
6year	0.785(0.768-0.803)	66.4 (62.8, 70.0)	76.8 (76.3, 77.2)	76.5 (76.5, 76.5)	5.9 (5.3, 6.4)	99.1 (98.9, 99.2)
7year	0.785(0.770-0.800)	67.9 (64.8, 71.0)	75.5 (75.0, 76.0)	75.3 (75.3, 75.3)	7.5 (6.9, 8.0)	98.8 (98.6, 98.9)
8year	0.778(0.764-0.792)	65.4 (62.6, 68.2)	77.4 (76.9, 77.8)	76.9 (76.9, 76.9)	9.6 (9.0, 10.3)	98.4 (98.2, 98.5)
9year	0.776(0.763-0.789)	66.3 (63.8, 68.9)	76.1 (75.6, 76.6)	75.7 (75.7, 75.7)	11.0 (10.3, 11.6)	98.1 (97.9, 98.3)
10year	0.773(0.761-0.785)	65.2 (62.8, 67.6)	76.6 (76.1, 77.0)	76.0 (76.0, 76.0)	12.6 (11.9, 13.4)	97.7 (97.5, 97.9)
**DL**						
2year	0.94 (0.91-0.97)	86.84 (76.09, 97.59)	89.15 (88.80, 89.49)	89.15 (88.80, 89.49)	0.97 (0.64, 1.29)	99.98 (99.97, 100)
3year	0.87 (0.86-0.89)	85.71 (80.82, 90.61)	73.68 (73.19, 74.17)	73.75 (73.26, 74.24)	2.02 (1.72, 2.32)	99.88 (99.83, 99.92)
4year	0.84 (0.82-0.86)	80.40 (76.50, 84.30)	71.79 (71.29, 72.29)	71.90 (71.40, 72.40)	3.55 (3.17, 3.94)	99.65 (99.57, 99.73)
5year	0.80 (0.79-0.82)	78.44 (75.20, 81.69)	67.02 (66.49, 67.55)	67.25 (66.73, 67.77)	4.58 (4.18, 4.98)	99.36 (99.25, 99.46)
6year	0.79 (0.77-0.80)	74.36 (71.45, 77.28)	69.37 (68.85, 69.89)	69.51 (69.00, 70.02)	6.56 (5.97, 6.94)	98.96 (98.82, 99.10)
7year	0.78 (0.77-0.79)	67.13 (64.41, 69.85)	75.70 (75.21, 76.18)	75.38 (74.91, 75.86)	9.54 (8.90, 10.18)	98.37 (98.21, 98.53)
8year	0.78 (0.76-0.79)	71.51 (69.19, 73.83)	70.93 (70.42, 71.45)	70.96 (70.46, 71.46)	10.73 (10.11, 11.35)	98.08 (97.89, 98.26)
9year	0.78 (0.77-0.79)	72.28 (70.18, 74.37)	70.27 (69.75, 70.79)	70.38 (69.88, 70.89)	12.64 (11.99, 13.30)	97.70 (97.50, 97.91)
10year	0.78 (0.77-0.79)	69.94 (67.96, 71.92)	73.06 (72.55, 73.57)	72.86 (72.36, 73.35)	15.50 (14.77, 16.24)	97.17 (96.95, 97.39)
