# Supplementary material for: Development and verification of prediction models for preventing cardiovascular diseases
Source: PLoS One. 2019 Sep 19;14(9):e0222809. doi: 10.1371/journal.pone.0222809 (PMC6752799; doi:10.1371/journal.pone.0222809)
Supplement: S3 Table — (PDF) [file pone.0222809.s006.pdf]

(a) Male

COX	AUC	Sensitivity	Specificity	Accuracy	PPV	NPV
2year	0.634(0.532,0.735)	94.7(84.7, 104.8)	32.3(30.0, 34.5)	33.0(33.0, 33.0)	1.6(0.9, 2.3)	99.8(99.5, 100.2)
3year	0.647(0.582,0.711)	87.0(78.1, 96.0)	37.9(35.5, 40.2)	39.4(39.4, 39.5)	4.4(3.2, 5.7)	98.9(98.1, 99.7)
4year	0.662(0.609,0.716)	72.3(63.3, 81.4)	53.0(50.5, 55.4)	54.0(54.0, 54.1)	8.3(6.4, 10.2)	97.0(95.9, 98.1)
5year	0.663(0.616,0.710)	70.8(63.0, 78.6)	53.4(50.9, 55.9)	54.7(54.7, 54.8)	11.3(9.1, 13.4)	95.6(94.3, 97.0)
6year	0.667(0.624,0.710)	67.5(60.2, 74.8)	56.4(54.0, 58.9)	57.5(57.4, 57.5)	13.7(11.3, 16.2)	94.4(92.9, 95.9)
7year	0.685(0.646,0.725)	70.8(64.3, 77.4)	57.3(54.8, 9.8)	58.8(58.8, 58.8)	17.0(14.3, 19.6)	94.1(92.6, 95.6)
8year	0.680(0.643,0.717)	69.5(63.5,75.6)	57.8(55.2,60.3)	59.3(59.3,59.3)	19.8(17.0,22.6)	92.7(91.0,94.4)
9year	0.681(0.646,0.716)	73.3(67.7,78.9)	54.3(51.8,56.9)	57.0(57.0,57.0)	20.7(18.0,23.5)	92.6(90.8,94.4)
10year	0.690(0.655,0.725)	70.4(64.8,76.1)	58.4(55.9,61.0)	60.2(60.2,60.2)	22.5(19.6,25.5)	92.0(90.3,93.8)
**DL**						
2year	0.869(0.826,0.912)	100(100,100)	71.9(69.8,74.1)	72.2(70.1,74.4)	3.9(2.2,5.6)	100(100,100)
3year	0.858(0.827,0.889)	100(100,100)	64.5(62.2,66.8)	65.7(63.4,67.9)	8.5(6.4,10.7)	100(100,100)
4year	0.853(0.829,0.878)	100(100,100)	65.8(63.5,68.2)	67.7(65.5,70.0)	14.7(12.0,17.5)	100(100,100)
5year	0.850(0.829,0.871)	100(100,100)	66.9(64.6,69.2)	69.5(67.3,71.7)	20.2(17.1,23.3)	100(100,100)
6year	0.853(0.833,0.873)	100(100,100)	67.3(64.9,69.7)	70.3(68.2,72.5)	23.9(20.6,27.2)	100(100,100)
7year	0.858(0.840,0.877)	99.5(98.4,100.5)	68.6(66.2,70.9)	71.9(69.8,74.1)	28.0(24.6,31.5)	99.9(99.7,100.1)
8year	0.851(0.831,0.871)	94.5(91.5,97.5)	69.4(67.0,71.7)	72.7(70.5,74.8)	31.7(28.1,35.2)	98.8(98.2,99.5)
9year	0.734(0.703,0.764)	83.1(78.3,87.8)	52.9(50.3,55.5)	57.1(54.8,59.5)	22.3(19.5,25.0)	95.0(93.5,96.5)
10year	0.764(0.735,0.792)	83.0(78.3,87.7)	57.7(55.2,60.3)	61.4(59.1,63.8)	25.2(22.2,28.2)	95.2(93.8,96.6)

(b) Female

COX	AUC	Sensitivity	Specificity	Accuracy	PPV	NPV
2year	0.621(0.480,0.764)	64.3 (39.2, 89.4)	64.1 (62.3, 66.0)	64.1 (64.1, 64.2)	1.0 (0.3, 1.6)	99.7 (99.4, 100)
3year	0.679(0.601,0.758)	44.9 (31.0, 58.8)	84.1 (82.7, 85.5)	83.3 (83.3, 83.3)	5.1 (3.0, 7.2)	98.8 (98.3, 99.2)
4year	0.701(0.640,0.762)	48.7 (37.4, 59.9)	84.5 (83.1, 85.9)	83.4 (83.4, 83.5)	8.6 (6.0, 11.3)	98.2 (97.7, 98.8)
5year	0.696(0.645,0.748)	72.1 (63.7, 80.4)	60.4 (58.5, 62.3)	60.9 (60.9, 60.9)	7.5 (5.9, 9.1)	98.0 (97.3, 98.7)
6year	0.716(0.673,0.759)	74.7 (67.6, 81.7)	61.0 (59.1, 62.9)	61.8 (61.7, 61.8)	10.2 (8.4, 12.0)	97.6 (96.8, 98.4)
7year	0.723(0.686,0.762)	73.9 (67.5, 80.3)	61.3 (59.4, 63.3)	62.2 (62.2, 62.2)	12.4 (10.4, 14.4)	96.9 (96.1, 97.8)
8year	0.727(0.692,0.762)	77.7(72.1,83.3)	57.4(55.5,59.4)	59.1(59.1,59.1)	13.8(11.9,15.8)	96.7(95.8,97.6)
9year	0.726(0.693,0.759)	78.1(72.8,83.4)	57.8(55.8,59.8)	59.6(59.6,59.6)	15.4(13.3,17.4)	96.4(95.5,97.4)
10year	0.725(0.694,0.756)	79.4(74.4,84.5)	56.6(54.6,58.6)	58.7(58.7,58.8)	16.1(14.0,18.2)	96.3(95.3,97.3)
DL						
2year	0.900(0.850,0.949)	92.9(79.4,106.3)	71.6(69.9,73.3)	71.7(70.0,73.5)	1.7(0.8,2.7)	99.9(99.8,100.1)
3year	0.852(0.821,0.884)	100(100,100)	65.0(63.2,66.9)	65.7(63.8,67.5)	5.2(3.8,6.6)	100(100,100)
4year	0.853(0.829,0.877)	100(100,100)	66.3(64.5,68.1)	67.3(65.5,69.1)	8.2(6.4,9.9)	100(100,100)
5year	0.855(0.834,0.876)	99.1(97.3,100.9)	66.2(64.4,68.1)	67.6(65.8,69.4)	11.5(9.5,13.6)	99.9(99.8,100.1)
6year	0.862(0.844,0.880)	100(100,100)	66.1(64.2,68.0)	68.0(66.2,69.8)	14.9(12.7,17.1)	100(100,100)
7year	0.866(0.850,0.883)	98.3(96.5,100.2)	68.0(66.2,69.9)	70.1(68.4,71.9)	18.6(16.1,21.0)	99.8(99.6,100)
8year	0.864(0.847,0.881)	96.7(94.3,99.1)	67.5(65.7,69.4)	69.9(68.1,71.6)	20.8(18.2,23.3)	99.6(99.3,99.9)
9year	0.769(0.742,0.796)	87.6(83.3,91.8)	54.9(52.9,56.9)	57.8(55.9,59.7)	16.0(14.0,18.0)	97.8(97.0,98.6)
10year	0.785(0.760,0.810)	78.6(73.5,83.7)	65.5(63.6,67.5)	66.8(65.0,68.6)	19.3(16.9,21.8)	96.7(95.8,97.6)
