# Supplementary material for: Development and verification of prediction models for preventing cardiovascular diseases
Source: PLoS One. 2019 Sep 19;14(9):e0222809. doi: 10.1371/journal.pone.0222809 (PMC6752799; doi:10.1371/journal.pone.0222809)
Supplement: S4 Table — (PDF) [file pone.0222809.s007.pdf]

| | Internal data set | | External data set | |
|---|---|---|---|---|
| | Male | Female | Male | Female |
| 2year | 0.753 (0.704-0.803) | 0.789 (0.705-0.872) | 0.634 (0.536,0.732) | 0.620 (0.484,0.757) |
| 3year | 0.742 (0.715-0.768) | 0.783 (0.749-0.817) | 0.645 (0.584,0.707) | 0.677 (0.599,0.754) |
| 4year | 0.749 (0.730-0.767) | 0.783 (0.757-0.808) | 0.659 (0.608,0.710) | 0.697 (0.637,0.758) |
| 5year | 0.758 (0.744-0.772) | 0.781 (0.761-0.802) | 0.660 (0.615,0.704) | 0.693 (0.642,0.743) |
| 6year | 0.759 (0.746-0.771) | 0.783 (0.766-0.800) | 0.663 (0.622,0.704) | 0.710 (0.669,0.752) |
| 7year | 0.752 (0.741-0.763) | 0.782 (0.767-0.797) | 0.678 (0.641,0.715) | 0.717 (0.680,0.754) |
| 8year | 0.751 (0.741-0.761) | 0.775 (0.762-0.789) | 0.675(0.641,0.710) | 0.720(0.685,0.754) |
| 9year | 0.750 (0.741-0.760) | 0.772 (0.760-0.785) | 0.675(0.641,0.708) | 0.718(0.685,0.750) |
| 10year | 0.747 (0.739-0.756) | 0.769 (0.758-0.781) | 0.677(0.644,0.710) | 0.715(0.683,0.747) |
