# Supplementary material for: Development and verification of prediction models for preventing cardiovascular diseases
Source: PLoS One. 2019 Sep 19;14(9):e0222809. doi: 10.1371/journal.pone.0222809 (PMC6752799; doi:10.1371/journal.pone.0222809)

(a) Internal data set (Male)

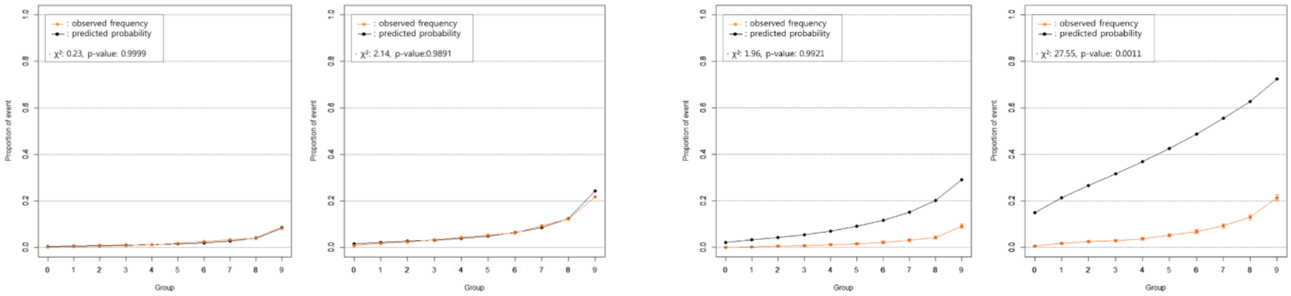

(b) Internal data set (Female)

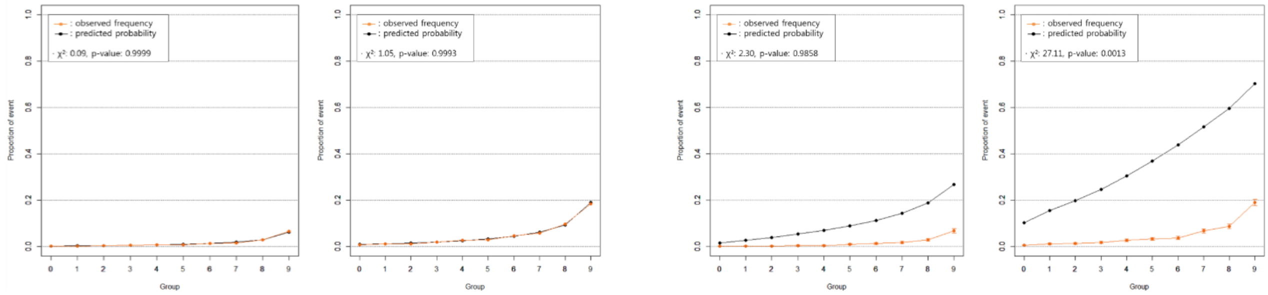

(c) External data set (Male)

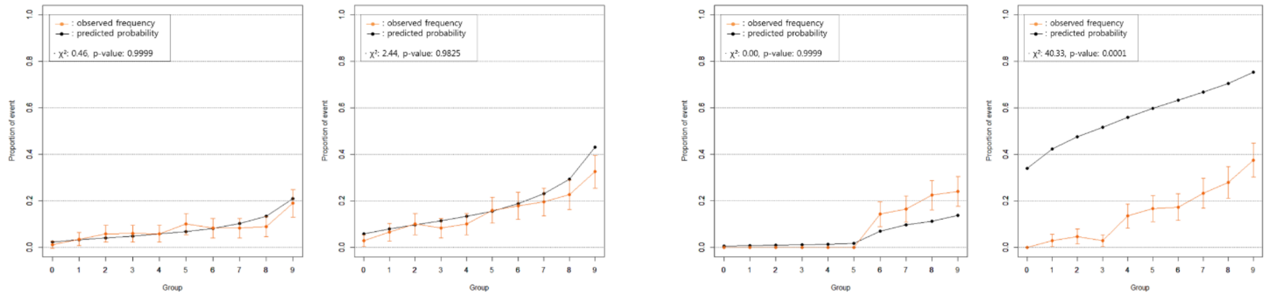

(d) External data set (Female)

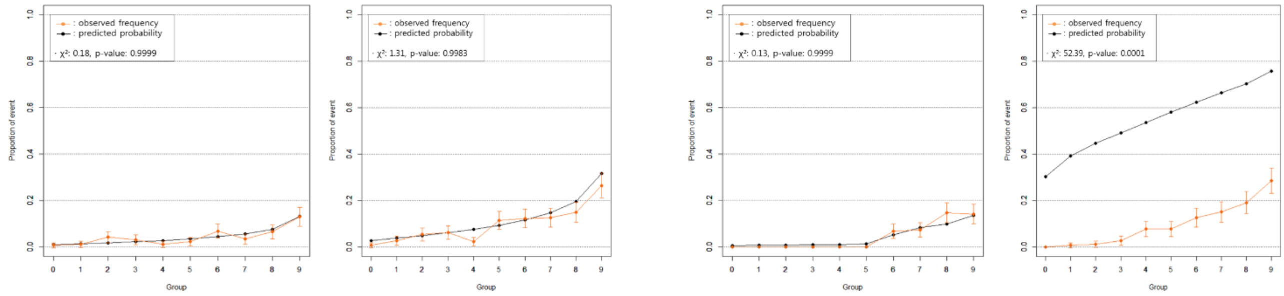

Supplement: S1 Fig — The left-hand figures represent 5-years and 10-years for the Cox regression model. The right-hand figures represent 5-years and 10-years for the DL model. (PDF) [file pone.0222809.s008.pdf]
