# Supplementary figures and images for: Development and verification of prediction models for preventing cardiovascular diseases
Source: PLoS One. 2019 Sep 19;14(9):e0222809. doi: 10.1371/journal.pone.0222809 (PMC6752799; doi:10.1371/journal.pone.0222809)

(a) Internal data set

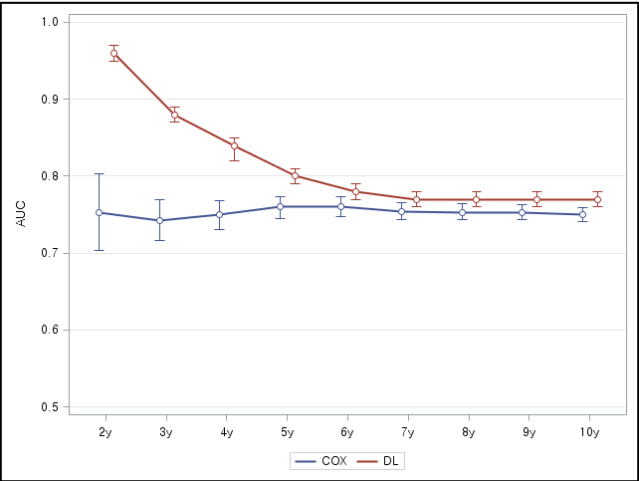

Male

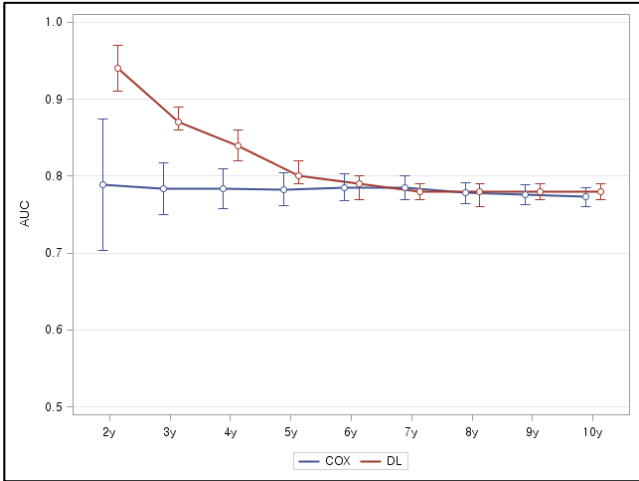

Female

(b) External data set

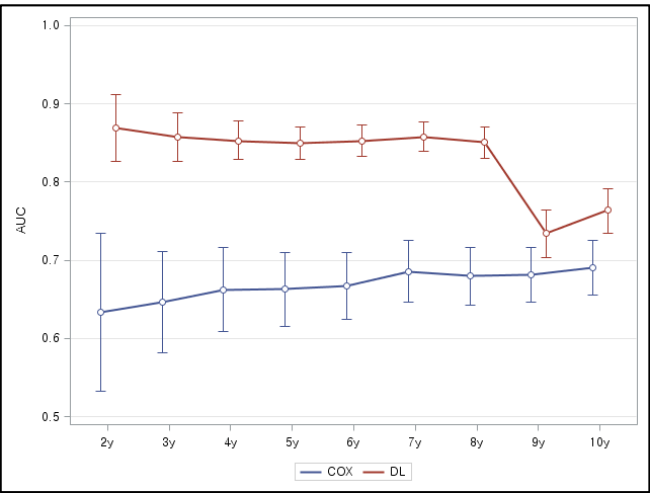

Male

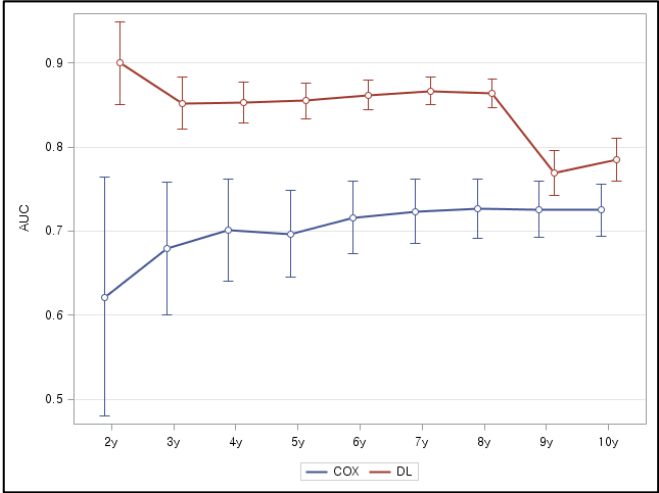

Female

Supplement: S2 Fig — (PDF) [file pone.0222809.s009.pdf]
